# Impact of concurrent medications on clinical outcomes of cancer patients treated with immune checkpoint inhibitors: analysis of Health Insurance Review and Assessment data

**DOI:** 10.1007/s00432-024-05728-z

**Published:** 2024-04-10

**Authors:** Soojung Hong, Ju Hyun Lee, Ja Yoon Heo, Koung Jin Suh, Se Hyun Kim, Yu Jung Kim, Jee Hyun Kim

**Affiliations:** 1 Division of Oncology-Hematology, Department of Internal Medicine, National Health Insurance Service, Ilsan Hospital, Goyang, Republic of Korea; 2 Division of Hematology/Medical Oncology, Department of Internal Medicine, Seoul National University Bundang Hospital, Seongnam, Republic of Korea; 3 Department of Internal Medicine, Seoul National University College of Medicine, Seoul, Republic of Korea

**Keywords:** Immune checkpoint inhibitors, Non-small cell lung cancer, Urothelial carcinoma, Malignant melanoma, Survival

## Abstract

**Purpose:**

Medications regulating immune homeostasis and gut microbiota could affect the efficacy of immune checkpoint inhibitors (ICIs). This study aimed to investigate the impact of concurrent medications on the clinical outcomes of patients with cancer receiving ICI therapy in South Korea.

**Methods:**

We identified patients newly treated with ICI for non-small cell lung cancer (NSCLC), urothelial carcinoma (UC), and malignant melanoma (MM) between August 2017 and June 2020 from a nationwide database in Korea. The effect of concurrent antibiotics (ATBs), corticosteroids (CSs), proton-pump inhibitors (PPIs), and opioids prescribed within 30 days before ICI initiation on the treatment duration and survival was assessed.

**Results:**

In all, 8870 patients were included in the ICI cohort (NSCLC, 7,128; UC, 960; MM, 782). The patients were prescribed ATBs (33.8%), CSs (47.8%), PPIs (28.5%), and opioids (53.1%) at the baseline. The median overall survival durations were 11.1, 12.2, and 22.1 months in NSCLC, UC, and MM subgroups, respectively, since starting the ICI mostly as second-line (NSCLC and UC) and first-line (MM) therapy. Early progression was observed in 34.2% of the patients. Opioids and CS were strongly associated with poor survival across all cancer types. A high number of concurrent medications was associated with early progression and short survival. Opioid and CS use was associated with poor prognosis in all patients treated with ICIs. However, ATBs and PPIs had a cancer-specific effect on survival.

**Conclusion:**

A high number of concurrent medications was associated with poor clinical outcomes.

## Introduction

Immune checkpoint inhibitors (ICIs) have revolutionized cancer treatment by targeting the brakes of the immune system and restoring antitumor activity. The clinical indications for ICIs, one of the novel standard treatments for various cancer types, are expanding. In South Korea, ICIs have been prescribed for patients with non-small cell lung cancer (NSCLC) and urothelial carcinoma (UC) as second-line treatment and for patients with malignant melanoma (MM) as first-line treatment since 2017. However, ICIs do not yield positive responses in all patients, and a significant proportion of patients fails to show a favorable response to the treatment (Hopkins et al. 2017). Therefore, it is necessary to develop biomarkers to predict the treatment response and optimize the clinical outcomes for each patient.

Immune checkpoints disrupt the adaptive immunologic processes that lead to cytotoxic T-cell apoptosis. Recent research has highlighted the important role of the gut microbiota in the immune system, which may affect the response of cancer cells to ICIs (Gopalakrishnan et al. 2018). The gut microbiota is a complex ecosystem of microorganisms that reside in the human intestine and play a crucial role in various physiological processes, including developing and maintaining the immune system (Belkaid and Hand 2014; Hooper et al. 2012). Evidence suggests that altered gut microbiota negatively impacts patient survival outcomes, primarily through acquired resistance mechanisms (Routy et al. 2018). In particular, medications that affect the immune homeostasis and gut microbiota, such as antibiotics (ATBs), corticosteroids (CSs), proton-pump inhibitors (PPIs), and opioids, have been shown to affect the efficacy of ICIs (Colard-Thomas et al. 2023; Hussain et al. 2021; Sieber et al. 2022).

However, the impact of concurrent medications on the treatment outcomes of ICI-treated patients with cancer is not demonstrated well in population-based studies, as most previous studies are small-scale retrospective analyses of patients in clinical trials or single-center studies. To address this knowledge gap, this study aimed to investigate the impact of concurrent medications on the clinical outcomes, such as treatment duration and overall survival (OS), of patients with cancer receiving ICIs through the analysis of real-world large-scale data from a nationwide Korean database.

## Methods

### Data source

The National Health Insurance Service is a compulsory health insurance system that covers 97% of the Korean population. The Health Insurance Review and Assessment Service (HIRA) is a government organization that built an accurate claims review and medical quality assessment system. We obtained data from the HIRA database, including demographic information, diagnostic codes, medical practice items, and prescribed medications.

### Study population

The patients who were diagnosed with stage 4 cancer and newly treated with ICI for NSCLC, UC, and MM between August 2017 and June 2020 were selected from the HIRA database. Reimbursement for ICI as second-line treatment for NSCLC and UC and first-line treatment for MM was first started in Korea in August 2017. Patients with multiple primary cancers or those younger than 18 years were excluded. Cohort entry was defined as the first date of dispensing ICI.

### Medications of interest

Insurance-covered ICIs, namely pembrolizumab, nivolumab, and atezolizumab, were specifically investigated. Our objective was to evaluate the impact of concurrent medications on both treatment duration and OS. ATBs, CSs, PPIs, and opioids were the concurrent medications of interest. Concurrent medication use was defined as the prescription of any of these four medications within 30 days before ICI initiation. Patients who received any of these concurrent medications during this period were classified as “users,” while those who did not were classified as “non-users.” Furthermore, the number of concurrently used medications among these four drugs of interest was investigated to examine its relationship with the clinical outcomes. Patients were followed from the cohort entry date to minimize the risk of immortal time bias.

### Measures

A cohort study was conducted to examine the association between the use of concurrent medications and treatment outcomes in patients with metastatic NSCLC, UC, and MM treated with ICIs. The primary outcomes of interest were ICI treatment duration and OS. ICI treatment duration was calculated as the time from the first to the last claim date plus 21 days, considering that patients received ICI every 3 weeks. Early progression was defined as progression observed within 2 months of receiving ICI treatment (Park et al. 2021; Champiat et al. 2017; Ferrara et al. 2018). OS was calculated from the date of starting ICI to either the date of death or last follow-up. All patients were followed from the cohort entry until death or the end of the follow-up period (December 31, 2020). The baseline comorbidity 1 year before cohort entry was assessed to account for the medical conditions of the patients.

### Statistical analysis

Descriptive statistics were used to summarize patient characteristics; continuous variables were presented as frequencies and percentages for categorical variables and means (standard deviations) or medians (minimum–maximum). A logistic regression model was used to estimate the odds ratio (OR) and 95% confidence intervals (CIs) for early progression risk with concurrent medication use compared to non-use. A Cox proportional hazard model was used to estimate the hazard ratio (HR) and 95% CIs for mortality. Kaplan–Meier survival curves were used to estimate the OS and median survival time. Statistical significance was tested using a log-rank test. All statistical analyses were performed using SAS (version 9.4; SAS Institute, Cary, NC, USA).

## Results

### Cohort characteristics

**Fig. 1:**
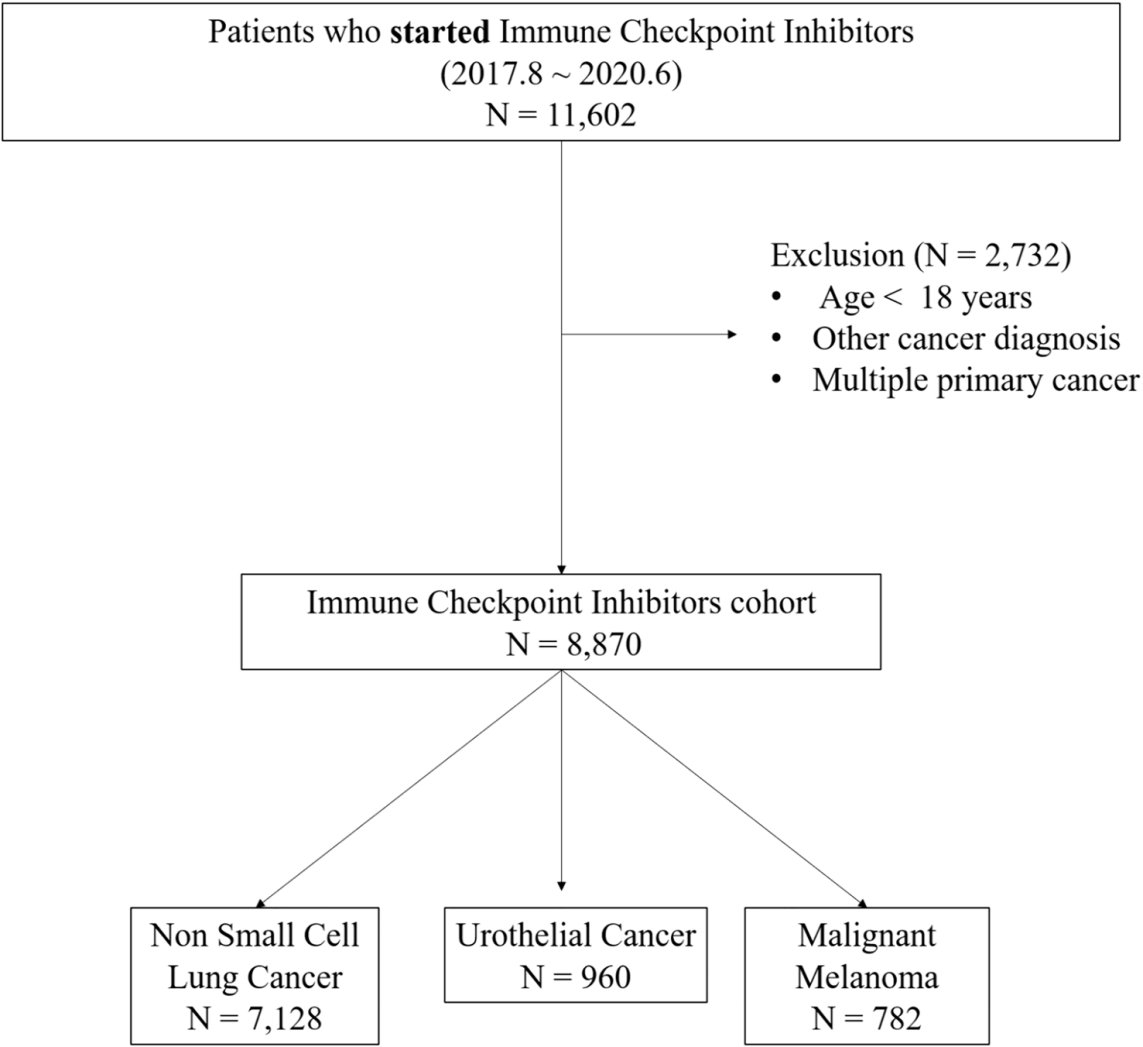
Selection of the study population

A total of 8,870 patients who met our inclusion criteria were identified. The ICI cohort consisted of 7,128 patients with NSCLC (80.4%), 960 with UC (10.8%), and 782 with MM (8.8%) (Fig. 1). Table 1 shows the baseline characteristics of the study population. The mean age of the ICI cohort was 66 ± 9.9 years, and two-thirds of the patients were male, but the male-to-female ratios were similar between the ICI and MM groups. One-third of the patients had fewer than two comorbidities.

**Table 1:** Baseline characteristics of the patients in the ICI cohort according to cancer type

	ICI cohort	NSCLC	UC	MM
Number of patients (%)	8870 (100.0)	7128 (80.4)	960 (10.8)	782 (8.8)
Age (years)				
Mean age (SD)	66 (9.9)	65.8 (9.5)	68 (10.1)	64.8 (12.5)
Age group: number (%)				
< 70 years	5409 (61.0)	4425 (62.1)	495 (51.6)	489 (62.5)
≥ 70 years	3461 (39.0)	2703 (37.9)	465 (48.4)	293 (37.5)
Sex: number (%)				
Male	6694 (75.5)	5569 (78.1)	714 (74.4)	411 (52.6)
Female	2176 (24.5)	1559 (21.9)	246 (25.6)	371 (47.4)
Type of comorbidity: number (%)				
Myocardial infarction	236 (2.7)	197 (2.8)	27 (2.8)	12 (1.5)
Congestive heart failure	1020 (11.5)	854 (12.0)	111 (11.6)	55 (7.0)
Peripheral vascular disease	1389 (15.7)	1098 (15.4)	166 (17.3)	125 (16.0)
Cerebrovascular disease	1354 (15.3)	1123 (15.8)	139 (14.5)	92 (11.8)
Dementia	193 (2.2)	127 (1.8)	43 (4.5)	23 (2.9)
Chronic pulmonary disease	6025 (67.9)	5250 (73.7)	472 (49.2)	303 (38.8)
Connective tissue disease-rheumatic disease	400 (4.5)	297 (4.2)	59 (6.6)	44 (5.6)
Peptic ulcer disease	3334 (37.6)	2726 (38.2)	382 (39.8)	226 (28.9)
Mild liver disease	3140 (35.4)	2520 (35.4)	358 (37.3)	262 (33.5)
Diabetes without complications	3365 (37.9)	2747 (38.5)	365 (38.0)	253 (32.4)
Diabetes with complications	1077 (12.1)	846 (11.9)	123 (12.8)	108 (13.8)
Paraplegia and hemiplegia	162 (1.8)	129 (1.8)	18 (1.9)	15 (1.9)
Renal disease	507 (5.7)	296 (4.2)	180 (18.8)	31 (4.0)
Moderate or severe liver disease	62 (0.7)	52 (0.7)	8 (0.8)	2 (0.3)
Number of comorbidities: number (%)				
0–1	2718 (30.6)	2058 (28.9)	297 (30.9)	363 (46.4)
2–3	3571 (40.3)	2979 (41.8)	339 (35.3)	253 (32.4)
4 or more	2581 (29.1)	2091 (29.3)	324 (33.8)	166 (21.2)
Type of ICI: number (%)				
Pembrolizumab	3502 (39.5)	2856 (40.1)	20 (2.1)	626 (80.1)
Nivolumab	2355 (26.6)	2145 (30.1)	54 (5.6)	156 (20.1)
Atezolizumab	3013 (34.0)	2127 (29.8)	886 (92.3)	0 (0)
Co-medications: number (%)				
Corticosteroids	4240 (47.8)	3758 (52.7)	308 (32.1)	174 (22.3)
Proton pump inhibitors	2529 (28.5)	2125 (29.8)	247 (25.7)	157 (20.1)
Opioids	4703 (53.0)	3854 (54.1)	491 (51.2)	358 (45.8)
Antibiotics	2995 (33.8)	2283 (32.0)	356 (37.1)	356 (45.5)
Broad spectrum	2816 (94.0)	2183 (95.6)	340 (95.5)	293 (82.3)
Narrow spectrum	179 (6.0)	100 (4.4)	16 (4.5)	63 (17.7)
Number of used co-medications: number (%)				
None	1696 (19.1)	1222 (17.1)	236 (24.6)	238 (30.4)
1	2552 (28.8)	2056 (28.8)	273 (28.4)	223 (28.5)
2	2499 (28.2)	2053 (28.8)	268 (27.9)	179 (22.9)
3	1575 (17.8)	1332 (18.7)	139 (14.5)	104 (13.3)
4	548 (6.2)	466 (6.5)	44 (4.6)	38 (4.9)
ICI treatment setting: number (%)				
1st line	887 (10.0)	173 (2.4)	28 (2.9)	686 (87.7)
2nd line	4970 (56.0)	4194 (58.8)	686 (71.5)	90 (11.5)
3rd line	1854 (20.9)	1649 (23.1)	201 (20.9)	4 (0.5)
≥ 4th line	1156 (13.1)	1112 (15.6)	45 (4.7)	2 (0.3)
Post-ICI treatment (number of administered medications): number (%)				
None	5443 (61.4)	4206 (59.0)	757 (78.9)	480 (61.48)
1	2283 (25.7)	1831(25.7)	169 (17.6)	283 (36.2)
2 or more	1144 (12.9)	1091 (15.3)	34 (3.5)	19 (2.4)
Palliative radiation therapy: number (%)				
Yes	1252 (14.1)	963 (13.5)	148 (15.4)	141 (18.0)
No	7618 (85.9)	6165 (86.5)	812 (84.6)	641 (82.0)

*ICI* immune checkpoint inhibitor, *MM* malignant melanoma, *NSCLC* non-small cell lung cancer, *UC* urothelial carcinoma

Although the three types of ICIs were used in similar proportions across the entire cohort, there were differences according to the cancer type and treatment setting. Atezolizumab was frequently prescribed to patients with UC (92.3%), while pembrolizumab (80%) and nivolumab (20%) were prescribed to patients with MM. Most patients with MM received ICIs as first-line treatment, with only 12% receiving ICIs as second or subsequent lines. Almost all patients with UC and NSCLC received ICIs after the first-line setting. More than half of the patients did not receive subsequent treatments after ICIs. Among all patients, 14.1% received palliative radiation therapy (RTx) during ICI treatment, with rates of 13.5% in NSCLC, 15.4% in UC, and 18.0% in malignant melanoma.

At the baseline, the patients were prescribed ATBs (33.8%), CSs (47.8%), PPIs (28.5%), and opioids (53.0%). Of the patients who received ATBs, 94% received broad-spectrum ATBs and only 6% received narrow-spectrum ATBs. About 20% of the patients did not use any of these four drugs, while 28% of the patients used one or two drugs in combination, and approximately 23% used three or four drugs in combination.

### Outcomes

The median (interquartile) follow-up duration was 7.5 (2.6–13.8) months, during which 4,773 (53.8%) deaths were reported. The median ICI treatment durations in the NSCLC, UC, and MM groups were 2.6 (0.8–43.5), 3.0 (0.8–37.5), and 4.6 (0.8–36.3) months, respectively. The overall median OS was 12.0 (95% CI 11.5–12.5) months, but it varied according to cancer type. The median survival time was the longest in the MM group (22.2 months), and the UC and NSCLC groups had similar median survival times (12.2 and 11.1 months, respectively).

**Fig. 2:**
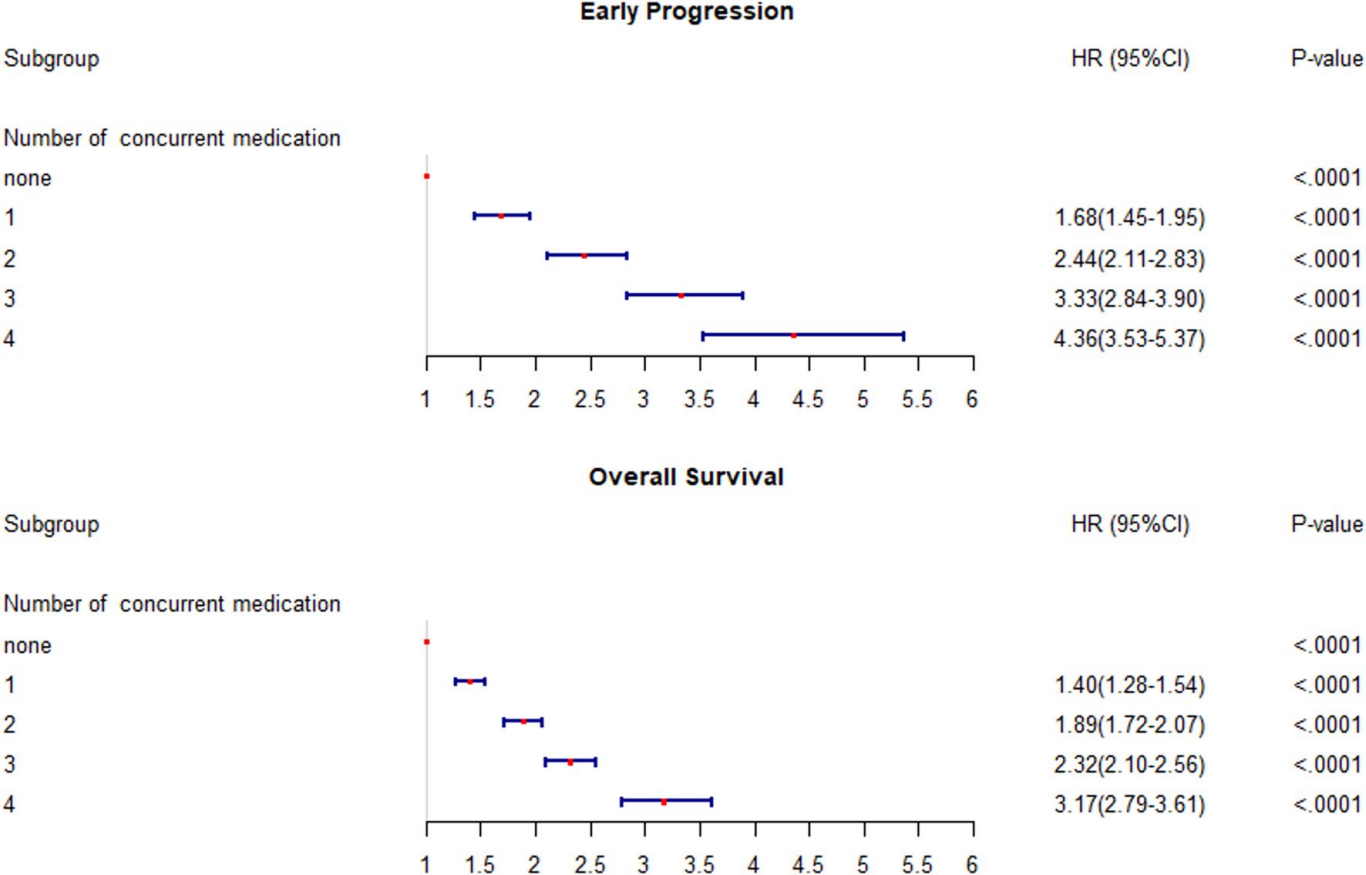
**a** Early progression and **b** overall survival risks according to the number of classes of concurrent medications of interest (antibiotics, corticosteroids, proton pump inhibitors, and opioids). *CI* confidence interval, *HR* hazard ratio

Early progressive disease (EPD) analysis was conducted using the data of the patients who met our defined criteria; the EPD rates were 34.2% for the total patient population and 36.2, 32.4, and 17.8% for NSCLC, UC, and MM groups, respectively. We assessed the factors that influence EPD for each cancer type (Table 2). In the multivariate analysis of patients with NSCLC, sex (OR for females, 1.14; 95% CI 1.01–1.29), ICI type (OR for atezolizumab vs. pembrolizumab, 1.56; 95% CI, 1.38–1.76), ATB use (OR, 1.50; 95% CI 1.35–1.68), CS use (OR, 1.54; 95% CI 1.39–1.69), opioid use (OR, 1.76; 95% CI 1.59–1.95), and ICI treatment setting (OR for third line or later, 1.18; 95% CI 1.07–1.31) had an impact on EPD. Among the patients with UC, CS use (OR, 1.49; 95% CI 1.11–2.01) and opioid use (OR, 2.80; 95% CI 2.07–3.83) had an impact on EPD, while for the patients with MM, only opioid use had an impact (OR, 1.78; 95% CI 1.19–2.68). On analyzing the number of concurrent medications, we found that the OR for EPD increased with an increase in the number of administered medications (Fig. 2). For palliative RTx, patients who received palliative RTx showed a benefit compared to those who did not receive RTx. The odds ratios were for 0.63 NSCLC, for 0.45 UC, and 0.52 for MM, respectively.

**Table 2:** Multivariate analyses for early progression

	NSCLC		UC		MM	
	OR (95% CI)	P-value	OR (95% CI)	P-value	OR (95% CI)	P-value
Age group		0.26		0.22		0.57
< 70 years	Ref		Ref		Ref	
≥ 70 years	1.06 (0.96–1.18)		1.20 (0.90–1.61)		1.12 (0.76–1.66)	
Sex		0.03		0.47		0.87
Male	Ref		Ref		Ref	
Female	1.14 (1.01–1.29)		1.13 (0.81–1.56)		0.97 (0.67–1.41)	
Number of comorbidities		0.33		0.24		0.62
0–1	Ref		Ref		Ref	
2–3	1.09 (0.96–1.23)	0.17	0.77 (0.54–1.11)	0.16	1.11 (0.73–1.71)	0.57
4 or more	1.03 (0.89–1.17)	0.80	1.00 (0.70–1.44)	0.98	0.89 (0.51–1.44)	0.63
Type of ICI		< 0.0001				
Pembrolizumab	Ref					
Nivolumab	1.13 (1.00–1.28)	0.05				
Atezolizumab	1.56 (1.38–1.76)	< 0.0001				
ICI treatment setting		0.001		0.44		
1st line and 2nd line	Ref		Ref			
≥ 3rd line	1.18 (1.07–1.31)		0.88 (0.63–1.22)			
Use of co-medications						
Antibiotics	1.50 (1.35–1.68)	< 0.0001	1.08 (0.80–1.47)	0.60	0.90 (0.60–1.35)	0.62
Corticosteroids	1.54 (1.39–1.69)	< 0.0001	1.49 (1.11–2.01)	0.01	1.18 (0.75–1.85)	0.47
Proton pump inhibitors	1.08 (0.97–1.21)	0.16	1.31 (0.95–1.81)	0.10	1.41 (0.89–2.23)	0.14
Opioids	1.76 (1.59–1.95)	< 0.001	2.82 (2.07–3.83)	< 0.0001	1.78 (1.19–2.68)	0.01
Palliative radiation		< 0.001		0.0004		0.02
No	Ref		Ref		Ref	
Yes	0.63 (0.54–0.74)		0.45 (0.29–0.69)		0.52 (0.3–0.92)	

*ICI* immune checkpoint inhibitor, *MM* malignant melanoma, *NSCLC* non-small cell lung cancer, *UC* urothelial carcinoma, *OR* odds ratio, *CI* confidence interval

**Fig. 3:**
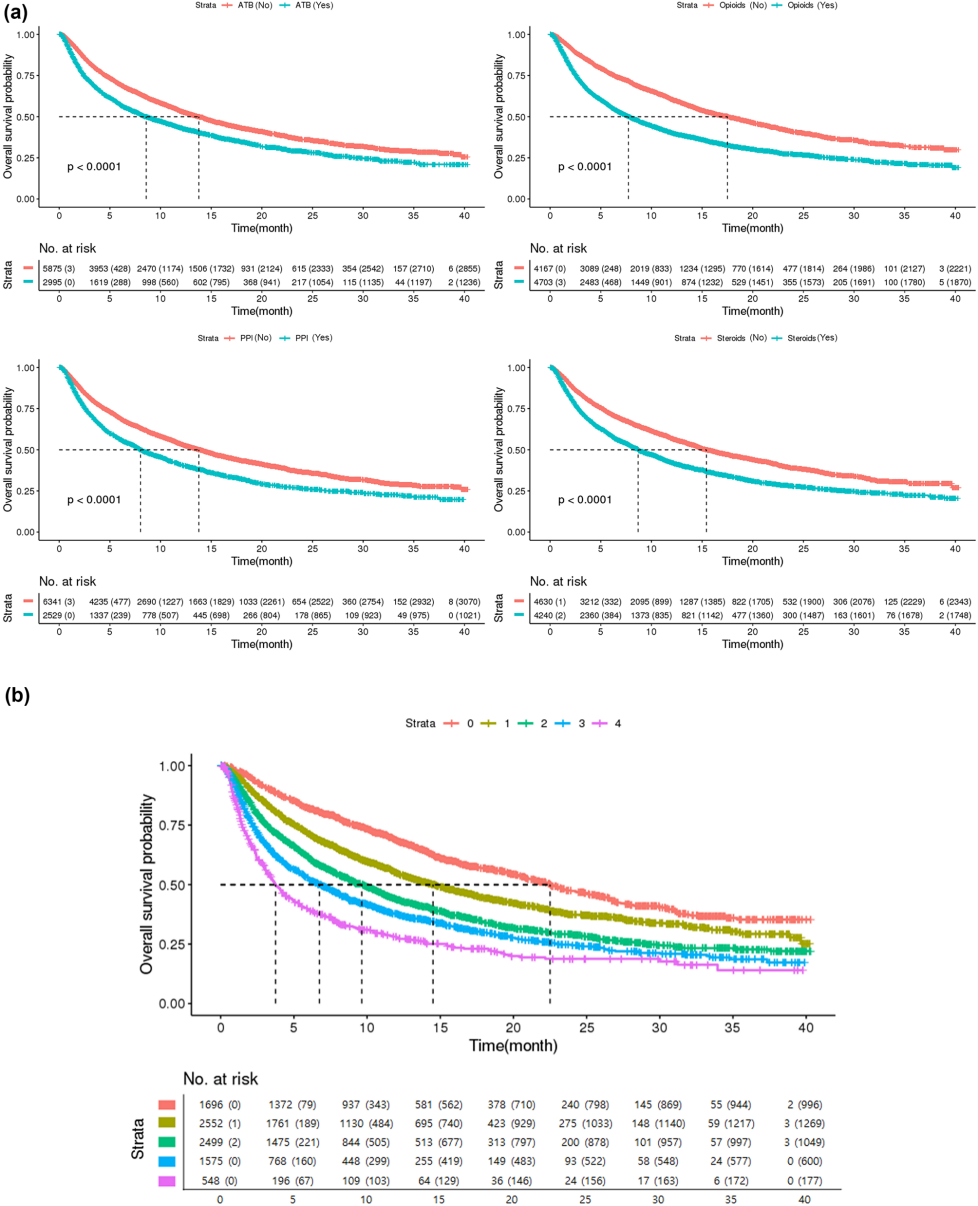
**a** Kaplan–Meier curves of each medication and **b** Kaplan–Meier curves based on the number of concurrent medications of interest (antibiotics, corticosteroids, proton pump inhibitors, and opioids)

Our multivariate survival analysis included age, sex, number of comorbidities, ICI type, ICI treatment setting, and four concurrent medications (ATBs, CSs, PPIs, and opioids). Opioids and CSs were strongly associated with poor survival across all three cancer types (Table 3). For patients with NSCLC, ATB and PPI use were also associated with poor OS (6.3 vs. 12.1 months; HR, 1.29; 95% CI 1.21–1.38 and 8.1 vs. 13.4 months; HR, 1.18; 95% CI 1.10–1.26, respectively). For patients with UC, the use of ATBs was associated with poor OS (8.1 vs. 12.6 months; HR, 1.24; 95% CI 1.03–1.50). However, the use of ATBs and PPIs did not affect survival in the MM group. Furthermore, in the NSCLC and MM groups, poor survival was observed when ICI was used as third or subsequent lines. Regarding number of concurrent medications, including ATBs, CSs, PPIs, and opioids, compared to non-use, the higher the number of administered medications, the shorter the survival duration of the patients (Table 4 and Figs. 2 and 3).

**Table 3:** Multivariate analyses for overall survival

	NSCLC		UC		MM	
	HR (95% CI)	P-value	HR (95% CI)	P-value	HR (95% CI)	P-value
Age group						
< 70 years	Ref		Ref		Ref	
≥ 70 years	1.15 (1.08–1.23)	< 0.0001	1.25 (1.05–1.50)	0.01	1.46 (1.16–1.82)	< 0.0001
Sex						
Male	Ref		Ref		Ref	
Female	0.96 (0.89–1.04)	0.34	0.90 (0.73–1.11)	0.33	0.88 (0.71–1.08)	0.22
Number of comorbidities		0.24		0.21		0.87
0–1	Ref		Ref		Ref	
2–3	1.07 (0.99–1.15)	0.10	0.86 (0.69–1.08)	0.19	1.08 (0.85–1.38)	0.54
4 or more	1.05 (0.97–1.15)	0.23	1.04 (0.83–1.30)	0.75	1.05 (0.79–1.40)	0.74
Type of ICI		< 0.0001				
Pembrolizumab	Ref					
Nivolumab	1.20 (1.11–1.29)	< 0.0001				
Atezolizumab	1.33 (1.23–1.44)	< 0.0001				
ICI treatment setting						
1st line and 2nd line	Ref		Ref		Ref	
≥ 3rd line	1.17 (1.10–1.25)	< 0.0001	1.11 (0.91–1.36)	0.31	1.56 (1.15–2.12)	< 0.0001
Use of co-medications						
Antibiotics	1.29 (1.21–1.38)	< 0.0001	1.24 (1.03–1.50)	0.02	0.79 (0.62–1.00)	0.05
Corticosteroids	1.32 (1.24–1.41)	< 0.0001	1.43 (1.19–1.72)	< 0.0001	1.38 (1.07–1.78)	0.01
Proton pump inhibitors	1.18 (1.10–1.26)	< 0.0001	1.22 (1.00–1.50)	0.05	1.27 (0.97–1.66)	0.08
Opioids	1.59 (1.49–1.70)	< 0.0001	1.68 (1.39–2.03)	< 0.0001	1.57 (1.23–1.99)	< 0.0001

*ICI* immune checkpoint inhibitor, *MM* malignant melanoma, *NSCLC* non-small cell lung cancer, *UC* urothelial carcinoma, *HR* hazard ratio, *CI* confidence interval

**Table 4:** Median overall survival duration according to the number of concurrent medications of different classes (antibiotics, corticosteroids, proton pump inhibitors, and opioids)

Number of concurrent medications	Median survival time (months) (95% confidence interval)		
	NSCLC	UC	MM
None	21.97 (20.2–23.59)	23.36 (16.25–NA)	26.3 (22.6–NA)
1	13.59 (12.63–14.9)	15.43 (11.58–20.23)	22.4 (17.24–NA)
2	8.85 (8.12–9.9)	9.84 (6.74–14.38)	21.9 (14.87–31.6)
3	6.61 (5.86–7.66)	4.77 (4.14–6.02)	15.2 (10.72–26.5)
4	3.55 (2.99–4.08)	3.65 (1.94–12.14)	10.5 (9.34–NA)

*MM* malignant melanoma, *NSCLC* non-small cell lung cancer, *UC* urothelial carcinoma

## Discussion

In this population-based study, we found that patients receiving a high number of concurrent medications with ICIs were at an increased risk of EPD and poor survival outcomes. Interestingly, the impact of these medications on EPD or OS varied according to the cancer type. For all the cancer types, opioid use was consistently identified as a strong predictor of EPD. Additionally, both opioid and CS use had substantially negative impact on OS.

ICIs inhibit the immune evasion mechanisms employed by cancer cells and promote immune responses against them. While ICI therapy has shown remarkable efficacy in subsets of patients, not all patients show a favorable response; thus, identifying the characteristics of patients who are likely to benefit from it (e.g., short treatment duration) is crucial. The gut microbiome has emerged as a potential factor contributing to the variability in ICI response (Colard-Thomas et al. 2023; Gopalakrishnan et al. 2018; Schirmer et al. 2016). Several previous studies have explored the effects of concomitant medication use on ICI treatment outcomes and suggested complex associations between the gut microbiome and immunotherapy responses and generally accepted the negative impact of CSs, ATBs, PPIs, and opioids on ICI efficacy (Colard-Thomas et al. 2023; Gaucher et al. 2021; Kalfeist et al. 2022; Weersma et al. 2020). Many studies have reported that ATBs directly disrupt the gut microbiome. Some studies have suggested that ATB use, particularly broad-spectrum ATBs, affects the gut microbiome that plays a role in modulating immune responses (Ahmed et al. 2018; Eng et al. 2023; Lu et al. 2021). Several meta-analyses have demonstrated that ATB use is associated with reduced response and decreased survival in patients receiving ICIs (Elkrief et al. 2019; Jiang et al. 2022; Tinsley et al. 2020; Wu et al. 2021; Yang et al. 2020; Yu et al. 2021). PPIs are the most frequently prescribed drugs to relieve digestive symptoms, and one study showed that more than a quarter of the patients with cancer receive PPIs (Raoul et al. 2021). Suppression of gastric acidity could increase the gastric PH, leading to a change in the gut microbiome and immune regulation. In addition to disturbing the gut microbiome, PPIs could directly impact the inflammatory response by reducing the secretion of adhesion molecules by inflammatory cells and inhibiting cytokine production (Hussain et al. 2021). Several studies suggest that PPI use may be associated with poor clinical outcomes in patients undergoing ICI therapy (Baek et al. 2022; Chalabi et al. 2020; Dar et al. 2022; Hopkins et al. 2022; Qin et al. 2021). Corticosteroids are potent immune-modulating agents that influence the secretion of various cytokines and play a role in T-cell activation, migration, and inhibition of differentiation (Kalfeist et al. 2022; Petrelli et al. 2020). They are commonly used by patients with cancer and transplant recipients, making immunosuppression-induced dysbiosis a topic of research in transplant settings (Colard-Thomas et al. 2023; Chong and Koh 2020). Some meta-analyses have reported negative effects of CSs on the survival of patients treated with ICIs (Petrelli et al. 2020; Zhang et al. 2021). It should be noted that patients requiring high doses of steroids, such as those with palliative reasons or brain metastases, may have pre-existing conditions that make them vulnerable to poor prognoses, which could be a confounding factor that cannot be excluded (Jessurun et al. 2021). Opioids are highly potent and frequently used analgesics in cancer therapy. However, many studies have demonstrated their potential to induce immune suppression through T-cell modulation and gut microbiome alterations (Prasetya et al. 2021). Preclinical studies have shown that opioids can inhibit certain immune cells, such as natural killer cells and T-cells, and impair their anti-tumor activity (Maher et al. 2019), resulting in concerns that opioid use may dampen the immune system’s ability to respond to ICI treatment. A few studies have suggested that opioid use is associated with poor clinical outcomes in patients receiving ICI therapy (Botticelli et al. 2021; Mao et al. 2022; Yu et al. 2022). However, it is important to interpret this in light of the fact that patients requiring opioids are likely to have relatively poor general conditions, aggressive disease, or a higher tumor burden.

In most previous studies, a limited sample size was used, and these four medications (ATBs, CSs, PPIs, and opioids) were individually evaluated for their impact on ICI efficacy. Using a model that combined ATB and CS use, Spakowicz et al. (2020) demonstrated that they had an additive effect on OS. Iglesias-Santamaría et al. (2020) investigated the use of ATBs and other concomitant medications, such as PPIs, CSs, and opioids. They suggested that the cumulative use of ATBs and concomitant opioids was associated with poor outcomes in patients undergoing ICI treatment. Additionally, Buti et al. (2021) found that their prognostic score calculated using three drug classes (ATBs, PPIs, and CSs) indicated progressively worsening outcomes with cumulative exposure to these drugs following ICI therapy. In contrast, we conducted our investigation using a large-scale population dataset to assess the impact of each of these four drugs, both individually and in combination, on the effectiveness of ICI therapy. Remarkably, as the number of concurrently administered medications increased, the efficacy of ICI treatment in patients appeared to be increasingly compromised. In comparison to the patients who did not use any of the four medications, those who used all four medications exhibited a 4.36-fold risk of EPD and a 3.17-fold risk of poor OS. Both CSs and opioids were consistently identified as independent poor prognostic factors for OS across all cancer types including NSCLC, UC, and MM. Generally, these medications are more frequently used in patients with advanced cancer who may have pre-existing conditions, high tumor burden, and pronounced symptoms, which could be the causes of poor outcomes. In the case of NSCLC, the use of ATBs and PPIs was associated with poor OS. However, the use of ATBs, not PPIs, negatively affected the OS in UC; the use of both ATBs and PPIs did not impact survival in the MM group. These divergent outcomes observed across the different cancer types may be attributed to the distinct biological characteristics and differences in the treatment lines or sequences specific to each cancer type.

Our study offers insights into the adverse impact of the use of concurrent medications on the clinical outcomes of patients receiving ICI treatment. However, determining a causal relationship in this study was challenging. It is important to acknowledge certain limitations of our study. Firstly, our study was based on claims data; therefore, information regarding the histologic type, clinical stage, and biomarkers such as PD-1, PD-L1, and tumor mutation burden were missing. In future research, it would be necessary to assess the clinical relevance of these pathological markers. Additionally, discussing the interaction between these biomarkers and concurrent medications would be beneficial. Additionally, despite being a population-based study, our study relied on retrospective data, which may have constrained our ability to control for confounding factors. Secondly, our study was limited to three specific cancer types, and further research is necessary to determine whether these findings can be extrapolated to other cancer types. Furthermore, the heterogeneity of the three cancer types and lack of standardization in the line of therapy for ICI use may have contributed to the complexity of our results. ICI utilization in South Korea adheres to the standardized insurance criteria, resulting in forced homogeneity within the patient population included in our claims data. This feature mitigates the drawbacks of our retrospective research. Another strength of our study is the concurrent assessment of all four drugs of interest.

In clinical practice, there are several considerations regarding the concurrent use of medications in cancer patients receiving ICI therapy. It's essential to recognize that introducing any new drug could have an unpredictable effect on expected oncologic outcomes. Based on our study findings, we recommend to adhere to evidence-based use of concurrent medications. For example, it is advisable to avoid prophylactic antibiotic use, opt for narrow-spectrum antibiotics whenever possible, and consider alternatives to proton pump inhibitors (PPIs), such as histamine H2-receptor antagonists, especially when initiating treatment. Corticosteroids, when used for supportive care in cancer patients, have been reported to have a more adverse impact on survival compared to their use for treating non-cancer-related conditions such as autoimmune diseases or chronic obstructive pulmonary disease (COPD) (Colard-Thomas et al. 2023). This could be associated with pre-existing poor conditions in cancer patients such as cord compression and brain metastasis. Similarly, in the case of opioids, patients requiring opioids are likely to have a higher tumor burden, more aggressive disease, and poorer general conditions. Our study did not establish a direct cause-effect relationship between concomitant medication and survival, however, it is highly advisable to take into account that for patients who need to continue high-dose corticosteroids or opioids for palliative purposes before starting ICI therapy, the expected effects of ICI treatment may not be achieved.

## Conclusion

The clinical outcomes of patients with cancer are adversely affected when ATBs, CSs, PPIs, and opioids are used either individually or concurrently with ICI, and these drugs have the potential to alter the composition of the gut microbiota. Although the causal relationship of these associations is not entirely clear, it is advisable for physicians to be aware that an increase in the number of drugs used tends to worsen the prognosis. Therefore, caution should be exercised when considering the use of these medications.

## Data Availability

The datasets generated during and/or analyzed during the current study are available from the corresponding author on reasonable request.
